# Supplementary material for: Causal Relationship between Aging and Anorexia Nervosa: A White-Matter-Microstructure-Mediated Mendelian Randomization Analysis
Source: Biomedicines. 2024 Aug 16;12(8):1874. doi: 10.3390/biomedicines12081874 (PMC11351342; doi:10.3390/biomedicines12081874)
Supplement: Supplementary file 1 [file biomedicines-12-01874-s001.zip › Supplementary Figure/Figure S9/Leaveoneout plot of MR analysis for ubm-b-1961 to AN.pdf]

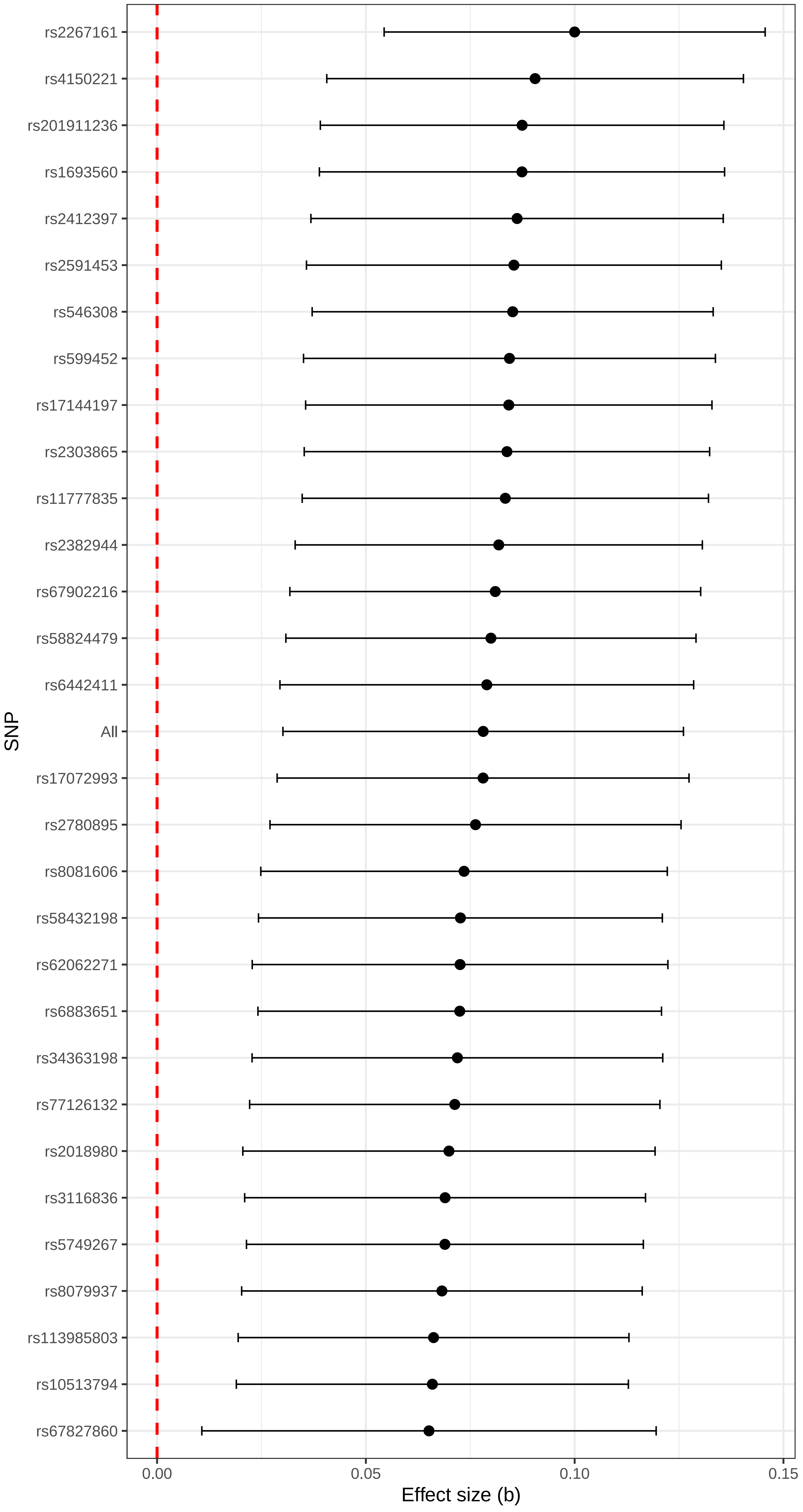

SNP
rs2267161
rs4150221
rs201911236
rs1693560
rs2412397
rs2591453
rs546308
rs599452
rs17144197
rs2303865
rs11777835
rs2382944
rs67902216
rs58824479
rs6442411
All
rs17072993
rs2780895
rs8081606
rs58432198
rs62062271
rs6883651
rs34363198
rs77126132
rs2018980
rs3116836
rs5749267
rs8079937
rs113985803
rs10513794
rs67827860
0.00
0.05
0.10
0.15
Effect size (b)
